# High Uterosacral Ligament Fixation Versus McCall's Culdoplasty for Vaginal Vault Suspension in Utero-Vaginal Prolapse Surgery

**DOI:** 10.7759/cureus.27368

**Published:** 2022-07-27

**Authors:** Aruna Verma, Monika Kashyap, Abhilasha Gupta

**Affiliations:** 1 Department of Obstetrics and Gynaecology, Lala Lajpat Rai Memorial (LLRM) Medical College, Meerut, IND; 2 Department of Obstetrics and Gynaecology, Venkateshwara Institute of Medical Sciences, Gajraula, IND

**Keywords:** pelvic organ prolapse quantification (pop-q), vault prolapse, levator ani muscle, total vaginal length(tvl), genital hiatus(gh), perineal body(pb), pop-q points, vaginal hysterectomy, vaginal high uterosacral ligament suspension, mccall's culdoplasty

## Abstract

Introduction: There are high chances of post-hysterectomy vault prolapse (PHVP) if the vault is not well supported after vaginal hysterectomy in cases of pelvic organ prolapse (POP). High uterosacral ligament suspension (HUSLS) and McCall's culdoplasty are the well-recommended modalities to suspend the vault after vaginal hysterectomy. As both the procedures are accessible to non-urologic gynaecologists, the study was planned in cases of POP.

Objective: The study was conducted to compare the anatomic and functional outcomes of patients undergoing vaginal HUSLS vs. McCall's culdoplasty at the time of vaginal hysterectomy.

Materials and methods: This prospective interventional study was done in a tertiary care hospital. A total of 80 patients were included and divided into two groups of 40 patients each. In one group, patients underwent high uterosacral ligament suspension and in the second group, McCall's culdoplasty was done for vault suspension. All procedures were done by two trained surgeons. The effectiveness of both the procedures was assessed by preoperative and postoperative pelvic organ prolapse quantification (POP-Q) (up to two years). Patients were followed for two years to see for any postoperative problem/recurrence.

Results: Vault suspension by HUSLS showed better results than McCall's culdoplasty, in terms of POP-Q point C, perineal body (PB), genital hiatus (GH) and total vaginal length (TVL) as compared to McCall's culdoplasty.

Conclusion: The anatomical correction is much better with HUSLS, which suspends the vault in the normal vaginal axis. However, it takes longer compared to McCall's culdoplasty, so the procedure should be individualised and performed with several precautions.

## Introduction

There is concern that hysterectomy, when performed for the indication of pelvic organ prolapse (POP), increases the risk of subsequent POP. So, prophylactic suspension of the vaginal cuff at the time of hysterectomy has been recommended to reduce the risk of vault prolapse [1]. The rationale for performing vaginal apical suspension with hysterectomy is to recreate the support provided by the cardinal and uterosacral ligament complexes (Level 1 support), thereby preventing future POP.

The risk of future prolapse appears to be increased when hysterectomy is performed for the indication of POP, while the risk of future POP repair in women with normal pelvic support is less clear [2]. A retrospective study carried out in a tertiary care hospital in the USA found that around 2,500 vaginal hysterectomies were performed in the center, but only 55% had a concomitant apical support procedure performed with hysterectomy [3].

Subsequently, the National Quality Forum recommended vaginal apical suspension at the time of hysterectomy to reduce the risk of POP in United States hospitals [4]; however, evidence-based based guidelines to choose the best surgical procedures for patients are not available. Thus, the success of surgery is defined as the patient's functional outcome, anatomical correction, and relief from symptoms.

The Green-top Guideline No. 46 of the Royal College of Obstetrics and Gynecologists states that high uterosacral ligament suspension (HUSLS) should only be offered as first-line management in females with POP [5]. The American Association of Gynecologic Laparoscopists has called for future research, specifically a randomized controlled trial comparing McCall's culdoplasty (with uterosacral plication) with vaginal HUSLS (without plication) [6]. This study was planned because both procedures are accessible to the nonurological surgeon.

## Materials and methods

Study design and period

This was a prospective interventional cohort study conducted from January 2018 to December 2020 at the Department of Obstetrics & Gynecology, Lala Lajpat Rai Memorial (LLRM) Medical College, Meerut, Uttar Pradesh, India, after obtaining clearance from the ethical committee (approval EC-1/2017/6579).

Study population

All women with pelvic organ prolapse who chose surgical treatment and were willing to participate were enrolled for the study. Written and informed consent was obtained before the procedure, after explaining all the risks and prognosis involved in the surgery. The patients were subjected to a detailed survey regarding their demographic data, duration of prolapse, and urinary and bowel symptoms which were affecting their quality of life. The patients were also asked detailed questions about their obstetric history, menstrual history, family history of prolapse, and history of precipitating factors such as coughing, constipation, and abdominal mass.

After a thorough general physical and systemic examination, POP quantification (POP-Q) staging was carried out. The patients who were willing to undergo surgery had the procedure explained to them. They underwent the relevant investigations for surgery and anesthesia. The patients chose whether to undergo conservative surgical procedures or hysterectomy with pelvic floor repair and vault suspension. The women who had chosen the option of hysterectomy were included in the study and underwent either HUSLS or McCall’s culdoplasty with concomitant hysterectomy. Randomization of patients was decided by the chit system.

High uterosacral ligament suspension is an intraperitoneal procedure that traditionally uses a permanent suture to suspend the vaginal apex to the remnant of the intermediate portion of the uterosacral ligament at the level of the ischial spine and cephalad with the incorporation of fibromuscular walls of the anterior and posterior vagina. We used delayed absorbable sutures, either number-1 polydioxanone (PDS II) or polygalactin-910 (Vicryl-1) for the procedure (both sutures manufactured by Ethicon, a subsidiary of Johnson & Johnson, Raritan, NJ, USA). After completion of the vaginal hysterectomy, a long moistened gauge was packed to keep the bowels away from the operative field, and appropriate retractors were used to expose the uterosacral ligament on each side. The ligament was pulled at its tied end to make it prominent and was caught by Allis forceps as close to the ischial spine as possible after palpating it digitally as well. One to two sutures were taken through the substance of the ligament rather than encircling it, avoiding ureteral involvement. The sutures were left long, and the same procedure was repeated on the opposite side. These sutures were tied to the endovaginal fascia and vaginal skin after the completion of colporrhaphy in the usual manner. A cystoscopy was done after every HUSLS before the closure of the vault.

The technique used for vaginal hysterectomy with McCall's culdoplasty is described by Raymond Lee of the Mayo Clinic [7]. After vaginal hysterectomy, one to two internal McCall's sutures were placed using Vicryl-1. External McCall's sutures were placed more cephalad to internal McCall's sutures and placed through the posterior vaginal wall .

A total of 80 patients were included in the study. Forty patients underwent vaginal hysterectomy with HUSLS, and 40 patients underwent vaginal hysterectomy with McCall's culdoplasty.

Several observations were made, including parity, age, BMI, surgery duration, and POP-Q points before and up to two years of surgery. If complications occurred during or after surgery, they were documented. A two-year follow-up was performed to see if there were any postoperative complications or recurrences.

Inclusion criteria

Inclusion criteria were all women with POP in reproductive, premenopausal, and postmenopausal age groups who were willing to undergo surgery; women who had given consent to participate in the study; and women with POP who were willing to undergo follow-up.

Exclusion criteria

Exclusion criteria were women with POP who were unfit for surgery; women who preferred to undergo conservative treatment; and women with a history of prior vault suspension.

Statistical analysis

The data was compiled and analyzed using MS Excel (R) Office 365 (Microsoft, Redmond, WA, USA), GraphPad Prism 8.4.2 (GraphPad Software, San Diego, CA, USA), and SPSS version 25 (IBM Corp., Armonk, NY, USA). Descriptive statistics were presented in the form of proportions/percentages for categorical variables and median/interquartile ranges along with mean and standard deviation for continuous data. Normalcy assessment was carried out for the distribution of data. Fisher’s exact test/chi-square test were used for the comparison of proportions (categorical variables) wherever necessary. Continuous variables were analyzed using the Mann-Whitney test along with U value (for independent group/unpaired data) and the Wilcoxon signed-rank test along with W scores (for paired data or the preoperative-postoperative comparisons). A P-value of < 0.05 was considered significant.

## Results

The present study included a total of 80 patients with pelvic organ prolapse; 40 patients underwent HUSLS, and for 40 patients vault suspension/repair was carried out with McCall's culdoplasty. The median age of patients in both groups was comparable (50 yrs vs. 48 yrs) (P-value 0.975). Median parity was compared and was found to be comparable (4 vs. 4) (P-value 0.9443). Median BMI in both groups was also comparable (21.60 vs. 21.40) (P-value 0.5999) (Table 1).

**Table 1:** Demographic Comparison HUSLS: high uterosacral ligament suspension

Age	HUSLS	McCall	P-value
Mean	48.65	49.23	0.9750
Standard deviation	10.55	9.07	U value
Standard error of the mean	1.67	1.43	796.50
Median	50.00	48.00	
Quartile 1	42.50	42.75	
Quartile 3	56.25	52.00	
Parity			P-value
Mean	4.20	4.15	0.9443
Standard deviation	1.51	1.17	U value
Standard error of the mean	0.24	0.18	792.50
Median	4.00	4.00	
Quartile 1	3.00	4.00	
Quartile 3	5.00	5.00	
BMI			P-value
Mean	22.29	21.83	0.5999
Standard deviation	2.67	1.92	U value
Standard error of the mean	0.42	0.30	745.00
Median	21.60	21.40	
Quartile 1	20.40	20.40	
Quartile 3	24.20	23.25	

Preoperative POP-Q scores for both groups were compared. It was observed that all POP-Q points and lengths were comparable without any statistically significant difference (Table 2). The effectiveness of HUSLS was assessed postoperatively by comparing the various POP-Q parameters preoperatively and postoperatively. It was observed that all parameters were improved significantly (P-value for Aa, Ba, C, genital hiatus [GH], total vaginal length [TVL], Aa, Bp was < 0.001, and 0.0133 for perineal body [PB]) in HUSLS group (Table 3). The effectiveness of McCall's culdoplasty was also assessed by comparing the various POP- Q parameters preoperatively and postoperatively. Points Aa, Ba, Ap, and Bp were improved significantly with McCall's culdoplasty (P-value < 0.0001), and GH was improved with a statistically significant difference (P-value 0.0004). Points C, PB, and TVL were not found to have much difference (P-values of points C, PB, and TVL were 0.2688, 0.6719, and 0.5328, respectively) (Table 4). In our study we found that vault suspension by HUSLS showed better results than McCall's culdoplasty, in terms of POP-Q points C, PB, GH, and TVL; however, the points Aa, Ba, Ap, and Bp were comparable without any significant difference (Table 5).

**Table 2:** Preoperative Comparison of HUSLS and McCall's Culdoplasty S.D.: standard deviation, SEM: standard error of mean, Q1: Quartile 1, Q3: Quartile 3, HUSLS: high uterosacral ligament suspension, GH: genital hiatus, PB: perineal body, TVL: total vaginal length

HUSLS vs. McCall	Pre-Op	Mean	S.D.	SEM	Median	Q1	Q3	P-value	U value
H	Aa	1.80	1.54	0.24	2.00	0.75	3.00	0.4521	725
M	Aa	1.60	1.65	0.26	2.00	0.75	3.00		
H	Ba	3.89	2.35	0.37	4.00	2.75	5.00	0.2625	684
M	Ba	3.16	2.57	0.41	4.00	1.00	4.63		
H	C	3.80	3.05	0.48	3.50	1.00	6.00	0.1728	658.50
M	C	2.40	4.32	0.68	3.00	-0.25	5.25		
H	GH	4.11	0.90	0.14	4.00	3.50	5.00	0.8381	765
M	GH	4.24	0.93	0.19	4.00	3.00	4.50		
H	PB	3.18	0.76	0.12	3.00	3.00	3.50	0.8736	784
M	PB	3.09	0.74	0.12	3.00	3.00	3.50		
H	TVL	7.94	1.14	0.18	8.00	7.38	8.50	0.2063	672
M	TVL	7.61	1.15	0.18	8.00	7.00	8.13		
H	Ap	-0.33	2.02	0.32	-1.00	-2.00	1.25	0.528	699
M	Ap	-0.28	1.95	0.19	-1.00	-2.00	1.00		
H	Bp	1.00	3.18	0.50	1.00	-2.00	3.00	0.118	653.50
M	Bp	1.26	2.99	0.42	1.00	-1.50	2.50		
H	D	-1.05	3.48	0.55	-2.00	-3.00	1.25	0.217	663.50
M	D	-1.36	3.55	0.56	-1.50	-2.50	1.50		

**Table 3:** Effectiveness of HUSLS (According to POP-Q) S.D.: standard deviation, SEM: standard error of mean, Q1: Quartile 1, Q3: Quartile 3, HUSLS: high uterosacral ligament suspension, POP-Q: pelvic organ prolapse quantification, GH: genital hiatus, PB: perineal body, TVL: total vaginal length

HUSLS	Pre vs. Post	Mean	S.D.	SEM	Median	Q1	Q3	P-value	W score
Pre	Aa	1.80	1.54	0.24	2.00	0.75	3.00	< 0.0001	-780.0
Post	Aa	-2.38	0.95	0.15	-3.00	-3.00	-2.00		
Pre	Ba	3.89	2.35	0.37	4.00	2.75	5.00	< 0.0001	-820.0
Post	Ba	-2.68	0.89	0.14	-3.00	-3.00	-3.00		
Pre	C	3.80	3.05	0.48	3.50	1.00	6.00	< 0.0001	-820.0
Post	C	-6.73	0.95	0.15	-7.00	-7.00	-6.00		
Pre	GH	4.11	0.90	0.14	4.00	3.50	5.00	< 0.0001	-402.0
Post	GH	3.34	0.76	0.12	3.00	3.00	4.00		
Pre	PB	3.18	0.76	0.12	3.00	3.00	3.50	0.0133	201.0
Post	PB	3.50	0.62	0.10	3.50	3.00	4.00		
Pre	TVL	7.94	1.14	0.18	8.00	7.38	8.50	< 0.0001	-405.0
Post	TVL	7.09	0.88	0.14	7.25	7.00	8.00		
Pre	Ap	-0.33	2.02	0.32	-1.00	-2.00	1.25	< 0.0001	-487.0
Post	Ap	-2.63	0.70	0.11	-3.00	-3.00	-2.00		
Pre	Bp	1.00	3.18	0.50	1.00	-2.00	3.00	< 0.0001	-603.0
Post	Bp	-2.68	0.62	0.10	-3.00	-3.00	-2.75		
Pre	D	-1.05	3.48	0.55	-2.00	-3.00	1.25	N/A	N/A
Post	D	-	-	-	-	-	-		

**Table 4:** Effectiveness of McCall's Culdoplasty (According to POP-Q) S.D.: standard deviation, SEM: standard error of mean, Q1: Quartile 1, Q3: Quartile 3, POP-Q: pelvic organ prolapse quantification, GH: genital hiatus, PB: perineal body, TVL: total vaginal length

McCall	Pre vs. Post	Mean	S.D.	SEM	Median	Q1	Q3	P-value	W Score
Pre	Aa	1.60	1.65	0.26	2.00	0.75	3.00	< 0.0001	-741.0
Post	Aa	-2.55	0.85	0.13	-3.00	-3.00	-2.00		
Pre	Ba	3.16	2.57	0.41	4.00	1.00	4.63	< 0.0001	-780.0
Post	Ba	-2.58	1.24	0.20	-3.00	-3.00	-3.00		
Pre	C	2.40	4.32	0.68	3.00	-0.25	5.25	0.2688	-52.0
Post	C	2.33	2.16	0.50	2.00	-0.50	-4.00		
Pre	GH	4.74	1.07	0.17	5.00	4.00	5.13	0.0004	-124.0
Post	GH	4.26	1.07	0.17	4.50	3.00	5.00		
Pre	PB	3.09	0.74	0.12	3.00	3.00	3.50	0.6719	-9.0
Post	PB	3.05	0.64	0.10	3.00	3.00	3.50		
Pre	TVL	7.61	1.15	0.18	8.00	7.00	8.13	0.5328	-26.0
Post	TVL	2.98	2.76	0.50	3.00	1.75	6.00		
Pre	Ap	-1.59	1.63	0.26	-2.00	-3.00	-1.00	< 0.0001	-285.0
Post	Ap	-2.63	0.67	0.11	-3.00	-3.00	-2.00		
Pre	Bp	-0.33	3.04	0.48	-1.00	-3.00	1.25	< 0.0001	-392.0
Post	Bp	-2.70	0.72	0.11	-3.00	-3.00	-2.00		
Pre	D	-2.50	3.69	0.58	-4.00	-5.00	-2.00	N/A	N/A
Post	D	-	-	-	-	-	-		

**Table 5:** Post-operative Comparison of HUSLS and McCall's Culdoplasty S.D.: standard deviation, SEM: standard error of mean, Q1: Quartile 1, Q3: Quartile 3, HUSLS: high uterosacral ligament suspension, GH: genital hiatus, PB: perineal body, TVL: total vaginal length

HUSLS vs. McCall	Post-Op	Mean	S.D.	SEM	Median	Q1	Q3	P-value	U value
H	Aa	-2.38	0.95	0.15	-3.00	-3.00	-2.00	0.3905	722
M	Aa	-2.55	0.85	0.13	-3.00	-3.00	-2.00		
H	Ba	-2.68	0.89	0.14	-3.00	-3.00	-3.00	0.8689	789
M	Ba	-2.58	1.24	0.20	-3.00	-3.00	-3.00		
H	C	-6.73	0.95	0.15	-7.00	-7.00	-6.00	< 0.0001	283.50
M	C	-4.66	3.16	0.50	-5.00	-6.00	-5.00		
H	GH	3.34	0.76	0.12	3.00	3.00	4.00	< 0.0001	403
M	GH	4.26	1.07	0.17	4.50	3.00	5.00		
H	PB	3.50	0.62	0.10	3.50	3.00	4.00	0.0026	505
M	PB	3.05	0.64	0.10	3.00	3.00	3.50		
H	TVL	7.35	0.88	0.14	7.25	7.00	8.00	< 0.0001	391.50
M	TVL	5.78	3.16	0.50	6.00	5.75	7.00		
H	Ap	-2.63	0.70	0.11	-3.00	-3.00	-2.00	0.8304	779
M	Ap	-2.63	0.67	0.11	-3.00	-3.00	-2.00		
H	Bp	-2.68	0.62	0.10	-3.00	-3.00	-2.75	0.9992	796.50
M	Bp	-2.70	0.72	0.11	-3.00	-3.00	-2.00		

The time required for HUSLS was statistically longer compared with repair by McCall's culdoplasty (median HUSLS 40 min vs. 25 min McCall's culdoplasty, P-value < 0.0001). Follow-up time was also comparable in both groups (10.38 months vs. 10.10 months). Ureteral kinking was noted in only one case (2.5%) with HUSLS compared with McCall's culdoplasty, in which no ureteral injury was seen. Cystoscopy, which was required following each HUSLS, was used to diagnose ureteric kinking. When no urinary spurt was observed from the right-sided ureteral opening following the HUSLS procedure, the ureteric guidewire was passed, which could not be negotiated. So the HUSLS stitch on the side was removed, and the procedure was repeated. The guidewire was then passed without any hindrance. With McCall's culdoplasty, three patients (7.5%) complained of dyspareunia compared with HUSLS, where no patient had dyspareunia. Anatomical correction (stage 0 to 1) by HUSLS was seen in 38 out of 40 patients (95% of cases) compared with 32 out of 40 patients (80% of cases) who underwent McCall's culdoplasty. During follow-up, only one patient presented with stage 2 prolapse after 18-19 months (2.5%).

## Discussion

Vault suspension is a critical step to be included during surgeries for pelvic organ prolapse. Post-hysterectomy vault prolapse (PHVP) can develop owing to the lack of vault suspension. PHVP mainly occurs because of damage to the Level 1 supports of the vagina [8]. In an attempt to evaluate the effect of hysterectomy on recurrent prolapse, a study of approximately 100,000 women undergoing POP surgery in California between 2005 and 2011 showed an almost 30% decreased risk of repeat POP repair in women who underwent simultaneous hysterectomy compared with those who did not [9].

The primary aim of POP surgery is to restore the normal vaginal anatomy, improvement in vaginal bulge symptoms, and maintenance of normal bowel, bladder, and sexual functions. Recurrence rates of PHVP are variable with different surgical procedures for vault suspension. Several studies are available that assess the efficacy of various procedures, such as sacrocolpopexy, sacrospinous fixation, mesh application, McCall, etc. However, there is a paucity of data in the literature which assesses the efficacy of the vaginal HUSLS procedure. Thus, we designed the study to assess the efficacy and complications in the follow-up of HUSLS and to compare it with McCall's culdoplasty. Forty cases for each procedure were taken and evaluated.

Demographic features in terms of age, parity, and BMI were comparable for both groups. Preoperative POP quantification was also comparable for both groups, which is similar to the study by Bhalerao et al. in 2017 [10]. In our study, a significant correction was seen with HUSLS for all POP-Q points and lengths, in contrast to the study by Bhalerao et al., which showed insignificant results in terms of TVL [10]. In a meta-analysis and systemic review of HUSLS, apical, anterior, and posterior compartments were successfully managed in 81.2%, 98.3%, and 87.41%, respectively [9].

Following McCall's culdoplasty, significant improvement was seen in all POP-Q points except points C, PB, and TVL. Two cohort studies showed that patients who had undergone the McCall procedure supported the vault up to three years postoperatively [11]. In the present study, points C, GH, PB, and TVL were improved more significantly by HUSLS than by McCall's culdoplasty (P-value < 0.05). This is in contrast to the study by Bhalerao et al. where comparable results were seen for all POP-Q points except point C, i.e., in that study the vault was placed higher by HUSLS [10].

Anatomical success with HUSLS was seen in 95% of the patients in our study, which is confirmed by a similar study [9]. The time required for HUSLS was statistically longer compared with McCall's culdoplasty (median time 40 mins vs. 25 mins), and complications were almost similar in both groups. These findings were similar to those in the study by Spelzini et al. in 2017 [12].

During follow-up, only one patient presented with bulging symptoms after 18-19 months (2.5%). The rest of the patients were asymptomatic during the follow-up period. Milani et al. conducted a study on 533 subjects whose follow-up was 32 months and with a dropout rate of 2.6%. The most common complication was ureteral kinking (2.6%). The recurrence rate was 13.7% with HUSLS, but reoperation was needed only in 1% of patients [13].

Many studies support that HUSLS repairs all vaginal defects, the vaginal vault is well supported, and the vaginal axis is restored, which prevents further recurrence of prolapse. If this procedure is done vaginally, the chances of ureteral injury have been reported up to 11% [9]. Thus, it is essential to perform a cystoscopy after each vaginal procedure to check the ureteral patency. However, a retrospective analysis of 22 cases who underwent laparoscopic HUSLS for pelvic organ prolapse showed no ureteral injury [14]. A clinical trial of the efficacy of uterosacral ligament suspension during hysterectomy for the prevention of POP is ongoing [15].

## Conclusions

Both HUSLS and McCall's culdoplasty support the vault effectively, but anatomical correction is much better with HUSLS, which suspends the vault over the levator ani with normal axis toward the sacrum. However, HUSLS takes more operative time compared with McCall's culdoplasty, as well as there being a slightly increased risk of ureteral complications. So, the procedure should be individualized and performed taking several precautions. Cystoscopy is mandatory after every HULSL procedure.
